# Veterinary Institutions in the Developing World: Current Status and Future Needs

**DOI:** 10.3201/eid1012.040791

**Published:** 2004-12

**Authors:** John J. McDermott

**Affiliations:** * International Livestock Research Institute, Nairobi, Kenya

**Keywords:** Veterinary Institutions in the Developing World: Current Status and Future Needs,

Veterinary institutions help improve animal health by providing training that will enhance livestock production and trade and protect public health. The increasing role of animals in emerging infectious diseases has emphasized the need to improve veterinary services and integrate them with public health services more effectively.

The World Organisation for Animal Health devoted its Scientific and Technical Review (Volume 23, No. 1, April, 2004) to addressing these weaknesses and the rapidly changing environment of veterinary services in developing countries. The issue consisted of an introduction and summary by the coordinator and contributions from 28 persons organized into six sections. The first section examines the relative roles of the public and private sectors and included two papers on economic frameworks and two papers on technical experiences in providing public services by the private sector. A second section emphasizes changing international sanitary and phytosanitary regulations and how veterinary services could help meet these new requirements. The third, fourth, and fifth sections detail experiences in providing services across the developing world. The most common experiences include using paraprofessional staff, providing public services by the private sector, conducting surveillance, and monitoring and controlling infectious diseases. A final section explores anticipated financial and institutional capacity, research, and professional training needs.

Examples provided in the book detail many innovations relevant to delivering health services, particularly for providing access for poor and marginalized persons. However, the examples do not provide an analysis of different experiences across countries and systems. Veterinary services have evolved and should respond to social, economic, and political realities. Guidance should be provided to the decision makers who want an analysis of what works under different circumstances.

From an infectious disease perspective, the focus is on promoting livestock production and trade, with less emphasis on public health and food safety issues. This emphasis is consistent with current veterinary policy and practice in most developing countries, where economic development is more relevant than public health concerns. The World Organisation for Animal Health, which is responsible for international animal and animal product trade standards under the World Trade Organisation, also focuses on this perspective.

The book reflects the constraints and broad mandates of veterinary services in developing countries and the potential short-term conflicts of interests between livestock commerce and public health. However, the book does not address links between veterinary and public health services, including common surveillance, information, and disease control systems. Because the book highlights the veterinary components of disease control systems, it provides an important resource to develop such systems in the future.

